# Alzheimer's amyloid‐β and tau protein accumulation is associated with decreased expression of the LDL receptor‐associated protein in human brain tissue

**DOI:** 10.1002/brb3.1672

**Published:** 2020-06-02

**Authors:** Claire E. Shepherd, Andrew J. Affleck, Anita Y. Bahar, Francine Carew‐Jones, Gillian Gregory, David H. Small, Glenda M. Halliday

**Affiliations:** ^1^ Neuroscience Research Australia Randwick, Sydney NSW Australia; ^2^ University of New South Wales Randwick, Sydney NSW Australia; ^3^ Menzies Research Institute University of Tasmania Hobart TAS Australia; ^4^ Brain and Mind Centre University of Sydney Sydney NSW Australia

**Keywords:** Alzheimer's disease, amyloid‐β, ELISA, receptor‐associated protein, tau

## Abstract

**Introduction:**

One of the major neuropathological features of Alzheimer's disease (AD) is the accumulation of amyloid‐β (Aβ) protein in the brain. Evidence suggests that the low‐density lipoprotein receptor‐associated protein (RAP) binds strongly to Aβ and enhances its cellular uptake and that decreased RAP expression correlates with increased Aβ production in animal models of AD.

**Methods:**

The current study examined whether RAP levels change in AD human brain tissue and whether they are related to the amount of AD pathology. RAP and NeuN levels were determined by Western blot, while low‐density lipoprotein receptor‐related protein 1 (LRP1), tau and Aβ levels were determined by ELISA in the temporal cortex of 17 AD and 16 control cases.

**Results:**

An increase in total Aβ and insoluble and soluble tau protein was observed in AD brain tissue. In contrast, RAP levels were significantly decreased in AD brain tissue compared to controls. Correlation analysis revealed that levels of RAP correlated with both total Aβ and soluble and insoluble tau levels. Neither LRP1 nor NeuN levels were significantly altered in AD brain tissue homogenates and did not correlate with Aβ or tau protein levels.

**Conclusion:**

Reduction in RAP may contribute to the accumulation and aggregation of Aβ in the AD brain.

## Graphical abstract

Low‐density lipoprotein receptor‐associated protein (RAP) binds strongly to Aβ and enhances its cellular uptake and that decreased RAP expression correlates with increased Aβ production in animal models of AD. The current study examined whether RAP levels change in AD human brain tissue and whether they are related to the amount of AD pathology. RAP levels were significantly decreased in AD brain tissue compared to controls and correlated with both total Aβ and soluble and insoluble tau levels.

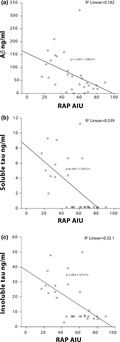

## 1 INTRODUCTION

Increased aggregation of Aβ occurs in the brain of all patients with Alzheimer's disease (AD) and is considered an important step in the disease pathogenesis. There is substantial evidence that certain components of the low‐density lipoprotein (LDL) receptor family and their ligands are involved in Aβ aggregation and/or clearance (Cam, Zerbinatti, Li, & Bu, 2005; Kanekiyo & Bu, 2014; Lane‐Donovan, Philips, & Herz, 2014; Sagare, Deane, & Zlokovic, 2012; Ulery et al., 2000; Zlokovic, Deane, Sagare, Bell, & Winkler, 2010). In particular, the low‐density lipoprotein receptor‐related protein 1 (LRP1) plays an important role in Aβ degradation and transport across the blood–brain barrier (Kanekiyo et al., 2011, 2013; Liu et al., 2017; Ma et al., 2018; Shibata et al., 2000; Storck et al., 2016), although its ability to bind Aβ directly has been questioned (Yamada et al., 2008) and studies assessing AD brain tissue levels have yielded conflicting results (Kang et al., 2000; Matsui et al., 2007). More recent evidence suggests that one of LRP1's major ligands, the receptor‐associated protein (RAP), is capable of binding to Aβ and inhibiting its aggregation and toxicity (Kerr et al., 2010) as well as enhancing its cellular uptake (Kanekiyo & Bu, 2009), thereby playing a potentially important role in Aβ aggregation (Van Uden et al., 2002; Xu et al., 2008). Indeed, RAP deficiency increases Aβ deposition in murine transgenic models of AD (Xu et al., 2008) and reduces Aβ clearance (Van Uden et al., 2002), and a previous immunohistochemical study has demonstrated decreased RAP immunohistochemistry in AD brain tissue (John Provias & Jeynes, 2010). The aim of the present study was to quantify protein levels of RAP in AD brain tissue and determine the relationship between RAP and major AD pathologies (Aβ and tau) and cell loss (NeuN). Our data demonstrate a significant decrease in RAP but not LRP1 in AD brain tissue. RAP, but not LRP1, levels were inversely associated with increases in Aβ and tau protein levels but no association with NeuN was identified. This strong association between RAP and AD pathologies lends further weight to a role for RAP in AD pathogenesis.

## 2 METHODS

### 2.1 Case characterization and tissues

Fresh‐frozen brain tissue from the inferior temporal cortex was provided from the Sydney Brain Bank for 33 cases, including 17 AD and 16 pathological and disease‐free controls, (Table 1). Consent for serial standardized assessments and autopsy, and the use of tissue for research purposes were obtained for all cases, and the brain donor program and tissue banking were approved by the Human Research Ethics Committee of the University of New South Wales. Neuropathology was confirmed using systematic assessments, as previously described (Shepherd, McCann, Thiel, & Halliday, 2002). Cases with head injury, significant cerebrovascular disease including stroke, and nontau intracellular inclusions or substantive neurodegeneration in the absence of intracellular inclusions were excluded. Alzheimer's disease cases met criteria for a high likelihood of AD (Montine et al., 2012). Disease‐free controls were cases without neurological or neuropsychiatric disorders and without significant neuropathology.

**TABLE 1:** Group demographics for cohorts

	Control	AD
Age at death (years)	74 ± 8	78 ± 10
Postmortem interval (hrs)	31 ± 17	27 ± 24
Disease duration (years)	‐	7 ± 3

Note

Group values are expressed as mean ± standard deviation.

### 2.2 Protein extraction

Crude protein extracts were prepared from 200 mg of frozen human brain tissue taken from the inferior temporal cortex for analysis of Aβ (Tris‐buffered saline (TBS) soluble, sodium dodecyl sulfate (SDS) soluble and formic acid soluble), NeuN (SDS soluble), RAP (SDS soluble), and LRP1 (SDS soluble). Our preliminary studies showed that NeuN, RAP, and LRP1 were located in the SDS fraction, so protein levels were analyzed in these fractions. Tissue was first homogenized in Tris buffer (20mM Tris‐HCl, 125mM NaCl, 5mM EDTA, 0.02% sodium azide) containing protease inhibitors (Roche), followed by centrifugation at 120,000 *g* for 2 hr at 4°C, with supernatant collected as the TBS‐soluble tissue fraction. The pellet was resuspended in TBS buffer containing 2% SDS and centrifuged at 120,000 *g* for 30 minutes at 25°C. The resulting membrane‐associated supernatant was retained as the SDS‐soluble fraction. The remaining pellet was resuspended in 70% formic acid (v/v) and centrifuged at 120,000 *g* for 1 hr at 4°C. The supernatant containing the insoluble proteins was retained as the insoluble fraction.

Tau was extracted as previously described (van Eersel et al., 2009). Briefly, tissue was homogenized in reassembly buffer (RAB) (0.75 M NaCl, 100 nM 2‐(N‐morpholino) ethanesulfonic acid, 1 mM EGTA, 0.5 mM MgSO_4_, 1mM dithiothreitol at pH 6.8, containing protease inhibitors, Roche). Homogenates were incubated on ice for 20 minutes while shaking and centrifuged at 100,000 *g* for 1 hr at 4°C. The resulting supernatant was retained as the RAB‐soluble protein fraction. The pellet was resuspended in paired helical fraction (PHF) extraction buffer (10 mM Tris, 10% sucrose, 0.85 M NaCl, 1 mM EGTA, pH 7.4) and centrifuged at 15,000 *g* for 20 minutes at 4°C. The supernatant was retained, and the pellet re‐extracted in PHF extraction buffer and centrifuged at 15,000 *g* for 20 minutes at 4°C. The supernatants were pooled and treated with 1% sarkosyl for 1 hr at 25°C prior to centrifugation at 100,000 *g* for 30 minutes. The pellet was resuspended in 50 mM Tris (0.2 ml/g of starting tissue, pH 7.4) constituting the sarkosyl‐insoluble protein fraction.

The protein concentrations of all fractions were measured using a bicinchoninic acid assay (Pierce BCA Protein Assay Kit, Thermo Scientific), according to the manufacturer's instructions. Samples were stored at −80°C until use.

### 2.3 Immunoblotting

Semiquantitative analysis of NeuN and RAP was determined by Western blot analysis. 25 μg of protein lysate was heated with sample buffer (2% SDS, 20% glycerol, 2.5% bromophenol blue, 12.5 mM Tris‐HCl, pH 6.8, 5% 2‐mercaptoethanol) and separated by reducing SDS‐PAGE gels before transfer to nitrocellulose membranes (Bio‐Rad). Antigen retrieval was performed on the NeuN membranes by boiling in 1x PBS (0.8% NaCl, 10.1 mM Na_2_HPO_4_·2H_2_O, 2.68 mM KCl, 1.76 mM KH_2_PO_4_, pH 7.4) for 1 minute on each side, and for the other membranes by boiling in 1x citrate buffer (Fronine) pH 6.0, for 3 minutes.

Membranes were blocked in 5% skim milk, washed in 1xTBS‐T (0.87% NaCl, 0.01 M Tris, pH 7.4, with 0.1% Tween‐20), and then incubated overnight in primary antibodies, which were mouse monoclonal anti‐NeuN (Millipore MAB377, 1:5,000 dilution) and mouse monoclonal anti‐LRPAP1 (Abcam ab20368, 1:6,000 dilution) with rabbit polyclonal GAPDH (Sigma‐Aldrich G9545, 1:4,000 dilution) used as a protein loading control. Protein detection was then performed using horseradish peroxidase‐conjugated secondary antibodies for mouse (Bio‐Rad #1,706,516) with enhanced chemiluminescence (Amersham ECL Plus Western Blot Detection System, GE Healthcare) or Alexa Fluor‐conjugated secondary antibodies for rabbit (Sigma‐Aldrich G9515).

A Bio‐Rad ChemiDoc MP system was used to capture images, and the relative levels of each protein of interest were analyzed using Image Lab software (Life Science Research, Bio‐Rad). The intensity of each protein band was quantified and expressed as arbitrary units standardized to GAPDH.

### 2.4 Enzyme‐linked immunosorbent assay (ELISA)

Analysis of Aβ was carried out using a Novex ABeta 42 and ABeta 40 Human ELISA kits (Life Technologies, Australia, VIC; cat # KHB3441 and KHB3481) according to the manufacturer's instructions. Soluble and insoluble tau proteins were analyzed using the Novex TAU (pS396) Human ELISA Kit (Life Technologies, Australia, VIC; cat # KHB7031). LRP1 analysis was carried out using a MyBiosource low‐density lipoprotein receptor‐related protein 1 (LDL‐RP) ELISA kit (Resolving Images, VIC, Australia; cat # MBS727011) according to the manufacturer's instructions.

### 2.5 Statistics

All statistical analyses were performed using SPSS software (version 21 for Mac, SPSS Inc.). A multilevel mixed model with RAP, LRP, NeuN, Aβ, and tau as the dependent variables was used to determine differences between control and AD groups. Age and postmortem delay were included as covariates. No significant effects of age (*p* > .08) or postmortem delay (*p* > .07) were identified so these variables were not considered further. Pearson's correlations were performed to determine relationships between variables. Aβ fractions were summed and analyzed as total Aβ (see Tables S1 and S2 for breakdown of fractional Aβ40 and Aβ42 levels, respectively). Probability (*p*) values <.05 were considered statistically significant.

## 3 RESULTS

### 3.1 Levels of RAP and LRP1 in the AD brain

**FIGURE 1:**
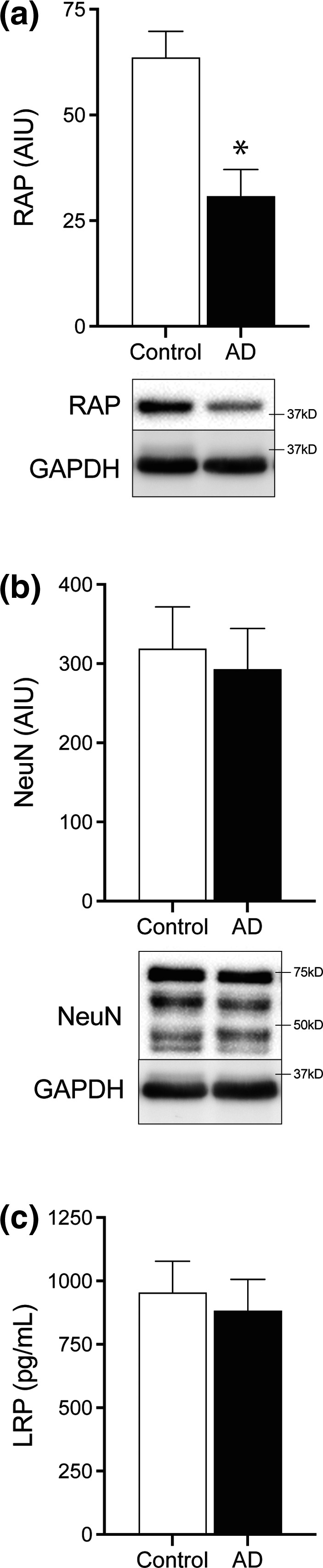
Bar graphs showing RAP (a), NeuN (b), and LRP1 (c) protein levels in AD and control brain tissue. RAP protein levels were significantly reduced in AD temporal cortex compared to controls (*p < .0001). No significant difference in LRP1 and NeuN protein levels was observed between groups. Error bars = standard error

Alzheimer's disease cases had significantly lower RAP levels compared to controls (*p* < .0001, Figure 1a) although no significant differences in the brain tissue levels of LRP1 (*p* = .45, Figure 1c) or NeuN (*p = *.59, Figure 1b) were seen between groups (Figure 1, Table 2).

**TABLE 2:** Estimated least squared means for assessed proteins

	Control	AD	*N* = C/AD
RAP (AIU)	63.6 ± 6.2	30.8 ± 6.3*	16/15
Aβ (ng/ml)	55.9 ± 27.7	152.0 ± 27.6*	15/15
Soluble tau (ng/ml)	0.1 ± 0.8	6.6 ± 0.8*	15/14
Insoluble tau (ng/ml)	4.8 ± 5.4	31.6 ± 5.2*	15/15
LRP (pg/ml)	954.8 ± 123.3	883.0 ± 123.3	12/15
NeuN (AIU)	319.1 ± 52.7	293.0 ± 51.3	16/17

Note

Group values are expressed as mean ± standard error. *p < .0001 versus controls. These means were estimated from a model setting age to 75 years and postmortem delay to 3 hr.

Abbreviations: AD, Alzheimer's disease; AIU, arbitrary volume intensity units; C, control; *N*, number of cases.

### 3.2 Relationships between brain protein levels

**FIGURE 2:**
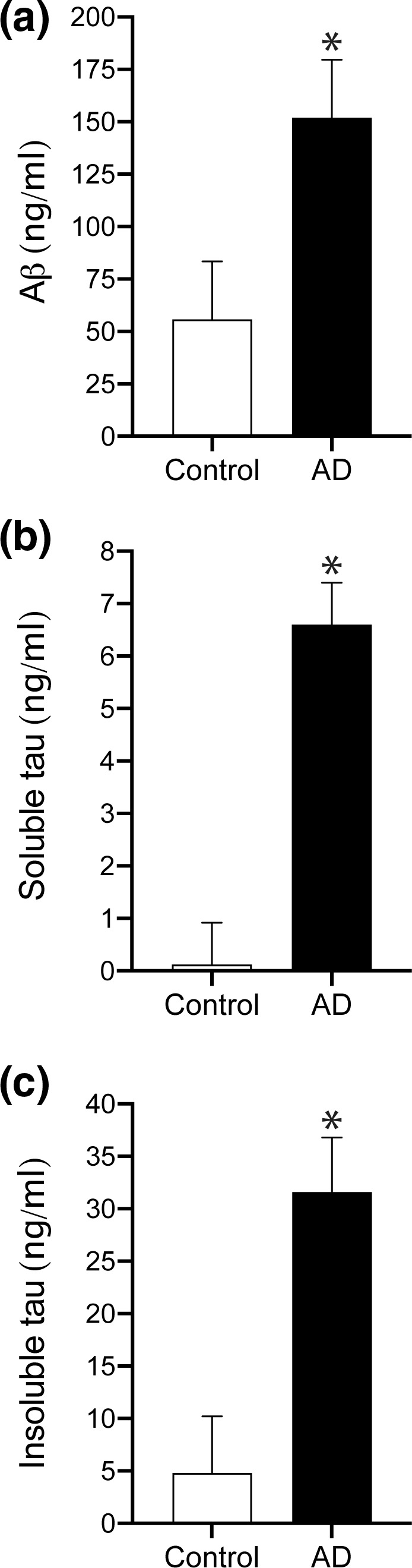
Bar graphs showing significantly elevated average total Aβ (a, *p* < .0001), soluble tau (b, *p* < .0001), and insoluble tau (c, *p* < .0001) protein levels in AD brain tissue compared to controls. Error bars = standard error

Consistent with neuropathological diagnosis, all AD cases had significantly greater Aβ and tau pathology compared to elderly controls. In particular, the levels of total Aβ (*p* < .0001, Figure 2a), soluble tau (*p* < .0001, Figure 2b), and insoluble tau (*p* < .0001, Figure 2c) were significantly higher in AD cases (Figure 2, Table 2).

As expected, Pearson's correlation analysis revealed significant positive associations between Aβ and both insoluble (*R* = .407, *p* = .028) and soluble (*R* = .775, *p* < .001) tau protein levels (data not shown).

**FIGURE 3:**
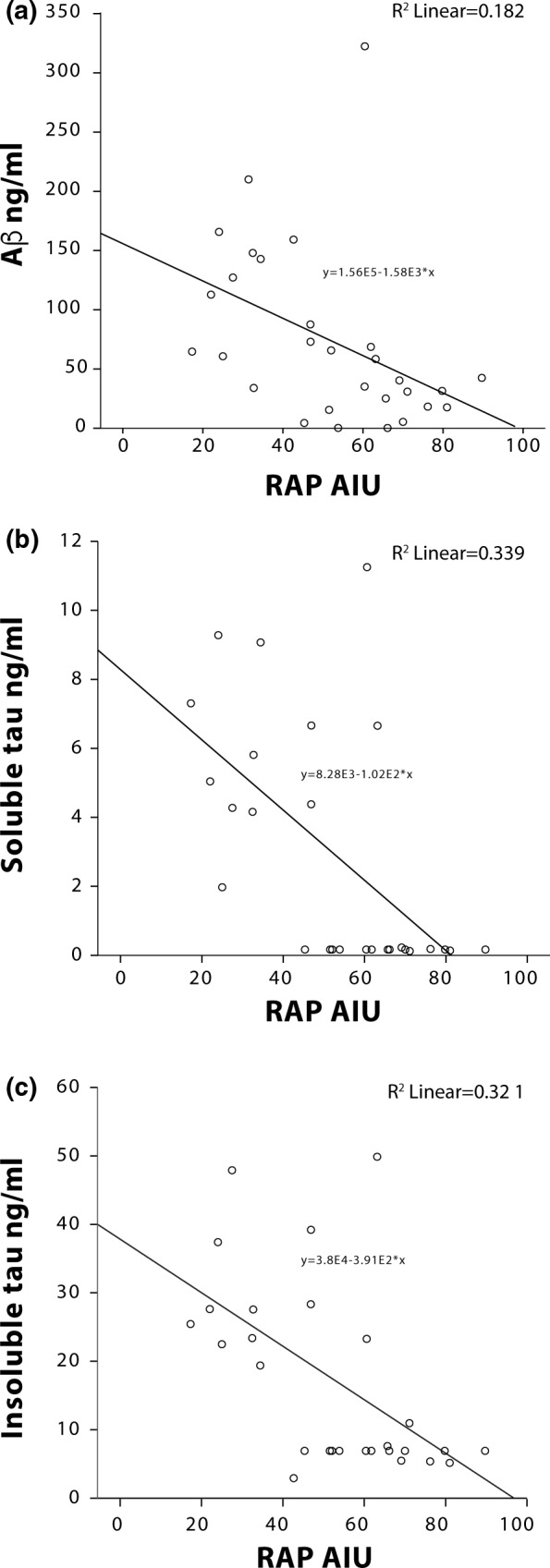
Scatter plot depicting the inverse relationship between RAP protein levels and total Aβ (a, R = −.426, *p* = .021), soluble tau (b, *R* = −.582, *p* = .001), and insoluble tau (c, *R* = −.566, *p* = .002) in AD brain tissue extracts

Levels of RAP correlated negatively with total Aβ (*R* = −.426, *p* = .021, Figure 3a), soluble tau (*R* = −.582, *p* = .001, Figure 3b), and insoluble tau (*R* = −.566, *p* = .002, Figure 3c), In contrast, LRP1 levels did not show any relationship with total Aβ or tau (*p* > .05).

## 4 DISCUSSION

Using biochemical methods, we show significantly reduced levels of RAP in AD brain tissue. Our data support a previous semiquantitative immunohistochemical study showing a decrease in the neuronal distribution of RAP in AD (Provias & Jeynes, 2010). We further show that in AD, low RAP levels are associated with high Aβ and tau (soluble and insoluble tau protein) pathologies, suggesting that RAP may play a role in the disease process and highlight the need for further research in this area.

As a chaperone protein, RAP is responsible for ensuring LRP1's effective transport to the cell surface (Bu & Schwartz, 1998) where it is known to play an important role in Aβ clearance (Sagare et al., 2012). RAP is also used as an effective LRP1 antagonist (Yamauchi et al., 2013), where it impacts cellular Aβ uptake (Yamada et al., 2008), indicating a mechanism whereby RAP can indirectly affect Aβ. Conversely, down‐regulation of RAP in AD transgenic mouse models has been shown to increase Aβ deposition (Xu et al., 2008), suggesting that this process is under tight homeostatic control. It has been widely speculated that increased Aβ deposition is primarily mediated by a decrease in the LDL receptor proteins, such as LRP1 and SorLA, as a consequence of decreased RAP. However, our data demonstrate a significant negative association between Aβ and RAP but not between Aβ and LRP1 in AD brain tissue. Furthermore, the ability of RAP to bind Aβ and act independently of LRP1 is supported by previous animal studies showing RAP deficiency does not significantly affect the levels of LRP1 (Xu et al., 2008), suggesting that either a relatively modest change in LRP1 may be significant enough to cause changes in Aβ deposition or RAP possesses an ability to affect Aβ independently of LRP1 (Xu et al., 2008). The latter hypothesis is supported by our previous studies demonstrating that RAP binds to Aβ and forms a complex (not necessarily colocalized with LRP1) on cell membranes (Kerr et al., 2010) and may be capable of enhancing the reuptake of Aβ in neuronal and glial cell cultures via an LRP1‐independent mechanism (Kanekiyo & Bu, 2009). It has also been shown that extracellular RAP does not inhibit the degradation of Aβ by neurons (by utilizing its affinity for LRP1), further indicating an LRP1‐independent mechanism of action (Narita, Holtzman, Schwartz, & Bu, 1997), which is not surprising given that many alternative Aβ lipid‐binding sites exist (Small et al., 2007; Subasinghe et al., 2003).

Despite a significant change in its chaperone protein, we did not identify a significant change in LRP1 in AD brain tissue or identify any association with Aβ or tau protein levels. This is not entirely surprising given previous studies have failed to report consistent changes in LRP1 in AD brain tissue, with some showing no change (Causevic, Ramoz, Haroutunian, Davis, & Buxbaum, 2003; Provias & Jeynes, 2014) and others showing increases (Matsui et al., 2007; Qiu, Strickland, Hyman, & Rebeck, 2001) or decreases (Kang et al., 2000) compared to controls. Cellular and regional variations (Kanekiyo & Bu, 2014), genetics (Kang et al., 2000), age at disease onset, and age at death (Kang et al., 2000) have all been proposed to affect LRP1 and may explain some of the previously reported variations. However, our findings in the temporal cortex do not support previous studies using the same region (Matsui et al., 2007) and we did not observe any association between LRP1 and age at onset (*p* = .541) or age at death (*p* = .196). While the present study did not specifically analyze changes in secreted LRP1, previous studies examining this fraction have also reported conflicting results (Causevic et al., 2003; Kang et al., 2000), thereby highlighting the complexity in this area and the need for additional research.

The relationship between RAP and tau pathology (soluble and insoluble tau) is less clear as no previous studies have investigated the interaction between these proteins either in vitro or in vivo. However, the strong relationship between Aβ and tau (Figure 2) suggests that any relationship with tau may be indirect. This is further supported by our observations of a lack of decrease in RAP protein expression in tauopathies such as frontotemporal lobar degeneration with tau deposition (FTLD‐tau vs. control RAP protein *p* = .144; see Figure S1). Interestingly, a combined genetic association between tau and LRP1 polymorphisms has previously been associated with a sixfold increased risk for AD, possibly via the alteration of intracellular cholesterol levels and tau phosphorylation (Vázquez‐Higuera et al., 2009), although we did not identify any association between LRP1 and tau in the present study. Two previous studies (Sánchez‐Guerra et al., 2001; Vázquez‐Higuera et al., 2011) have also failed to detect an association between single LRP1 or tau polymorphisms and AD, although the observation of reduced neurofibrillary tangle burden in statin‐treated AD patients postmortem (Li et al., 2007) suggests the relationship between tau and cholesterol pathways warrants further research.

Neuronal cell loss is a widely accepted feature of the AD brain. Its accurate measurement requires knowledge of the degree of atrophy, something which is difficult to do using isolated tissue samples of similar volumes. Hence, some studies report neuronal cell loss in medial temporal and neocortical regions at end‐stage AD (Andrade‐Moraes et al., 2013), while other studies report no significant cell loss, even in early affected hippocampal regions (Furcila, Dominguez‐Alvaro, DeFelipe, & Alonso‐Nanclares, 2019), supporting the notion that there is great variability in neuronal density in atrophied AD tissues (Arendt, Bruckner, Morawski, Jager, & Gertz, 2015). The findings in the present study were of a nonsignificant trend toward reduced NeuN in the same tissue volumes measured from the AD group compared to controls. This would be consistent with an overall neuronal loss in a region with tissue atrophy in AD.

## 5 CONCLUSION

Together with the evidence from both in vitro and in vivo animal studies, our observation that RAP is reduced in the AD brain and is significantly associated with increased Aβ pathology suggests that RAP plays an important role in the accumulation and aggregation of Aβ in AD, possibly via an LRP1‐independent mechanism. Further studies are required to elucidate any structural basis for RAP‐Aβ binding and to further our understanding of this complex cellular process.

## CONFLICT OF INTEREST

The authors have no conflicts of interest to declare.

## AUTHOR CONTRIBUTIONS

CS, DS, and GH participated in the experimental design and preparation of the manuscript; CS managed and planned all experimental procedures and analysis of the data; and GH and CS performed the statistical analysis. AB and GG performed the tau and Aβ ELISAs, FC‐J, AL, and AA performed the Western blots and data analysis and contributed to the preparation of the manuscript.

## Supporting information

Fig S1

Table S1‐S2

## Data Availability

The data that support the findings of this study are available from the corresponding author upon reasonable request.
