# Supplementary figures and images for: Alzheimer's amyloid‐β and tau protein accumulation is associated with decreased expression of the LDL receptor‐associated protein in human brain tissue
Source: Brain Behav. 2020 Jun 2;10(7):e01672. doi: 10.1002/brb3.1672 (PMC7375106; doi:10.1002/brb3.1672)

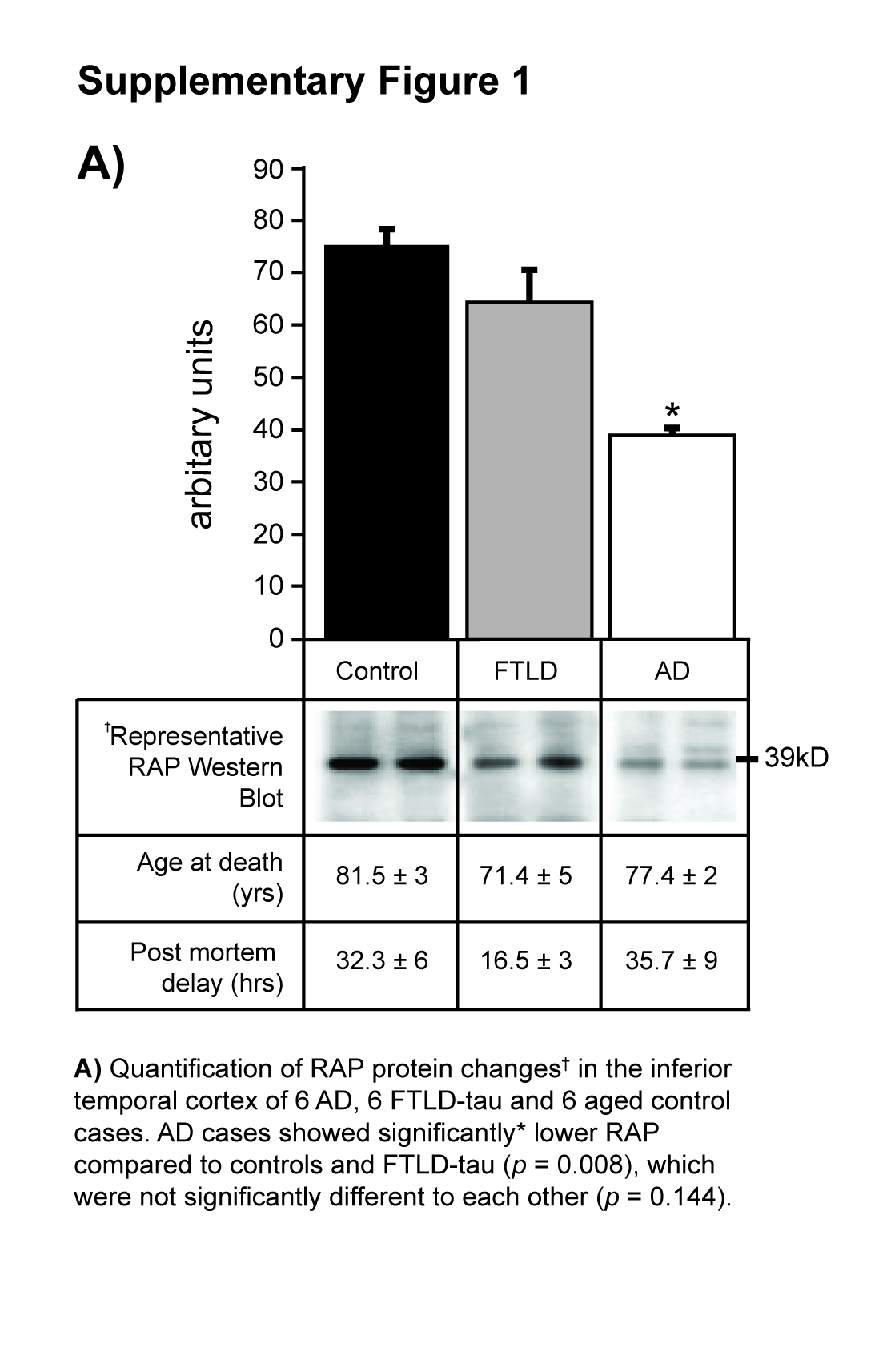

Supplement: Supplementary file 1 — Fig S1 [file BRB3-10-e01672-s001.tif]
