# Supplementary material for: Alzheimer's amyloid‐β and tau protein accumulation is associated with decreased expression of the LDL receptor‐associated protein in human brain tissue
Source: Brain Behav. 2020 Jun 2;10(7):e01672. doi: 10.1002/brb3.1672 (PMC7375106; doi:10.1002/brb3.1672)
Supplement: Supplementary file 2 — Table S1‐S2 [file BRB3-10-e01672-s002.docx]

**Supplementary Tables**

**Supplementary Table 1 – Aβ40 fraction levels for control and AD cases**

|  | Aβ40  soluble fraction | Aβ40  SDS fraction | Aβ40  formic acid fraction | Total  Aβ40 |
| --- | --- | --- | --- | --- |
| CONTROL | 1.4 ± 0.6 | 3 ± 1 | 3 ± 1 | 7 ± 1 |
| AD | 4 ± 2 | 22 ± 19 | 67 ± 45 | 93 ± 45 |

Soluble, SDS and formic acid Aβ40 expressed as a percentage of total Aβ40 summed for both AD and controls. Averages ± standard deviations

**Supplementary Table 2 – Aβ42 fraction levels for control and AD cases**

|  | Aβ42  soluble fraction | Aβ42  SDS fraction | Aβ42  formic acid fraction | Total  Aβ42 |
| --- | --- | --- | --- | --- |
| CONTROL | 4 ± 1 | 16 ± 10 | 12 ± 8 | 33 ± 10 |
| AD | 9 ± 4 | 25 ± 11 | 34 ± 10 | 67 ± 11 |

Soluble, SDS and formic acid Aβ42 expressed as a percentage of total Aβ42 summed for both AD and controls. Averages ± standard deviations
